# Supplementary material for: Reward recalibrates rule representations in human amygdala and hippocampus intracranial recordings
Source: Nat Commun. 2024 Nov 4;15:9518. doi: 10.1038/s41467-024-53521-w (PMC11535001; doi:10.1038/s41467-024-53521-w)
Supplement: Supplementary file 1 — Supplementary Information [file 41467_2024_53521_MOESM1_ESM.pdf]

Supplementary table 1. Number of trials in each condition for each of the contrasts.

Contrast	Mean number of trials (SD) Amygdala	Mean number of trials (SD) Hippocampus	Mean number of trials (SD) behaviour
Activity vs baseline	143 (18)	145 (15)	
Correct vs incorrect	60 (14)	61 (11)	
Shift vs no shift	59 (13)	60 (11)	
Shift correct vs no shift correct	27 (2)	28 (2)	
ID shift correct vs no shift correct	14(3)	14(4)	
ED shift correct vs no shift correct	14 (5)	15(5)	
No shift incorrect vs no shift correct	27 (5)	27 (5)	
Novel vs familiar	17 (3)	18 (3)	
ID shift vs no shift	25 (5)	26(4)	
ED shift vs no shift	35 (16)	35(15)	
Incorrect shift vs correct shift	25 (4)	26 (4)	
ED correct vs ED incorrect	15 (5)	15(5)	
ID correct vs ID incorrect	11(2)	11(2)	
Shift incorrect vs no shift incorrect	25 (6)	26 (6)	
ED RT			37(18)
ID RT			27(5)
No Shift RT			84(5)
ED errors			68(9)
ID Errors			63(12)
Set Change Errors			20 (5)

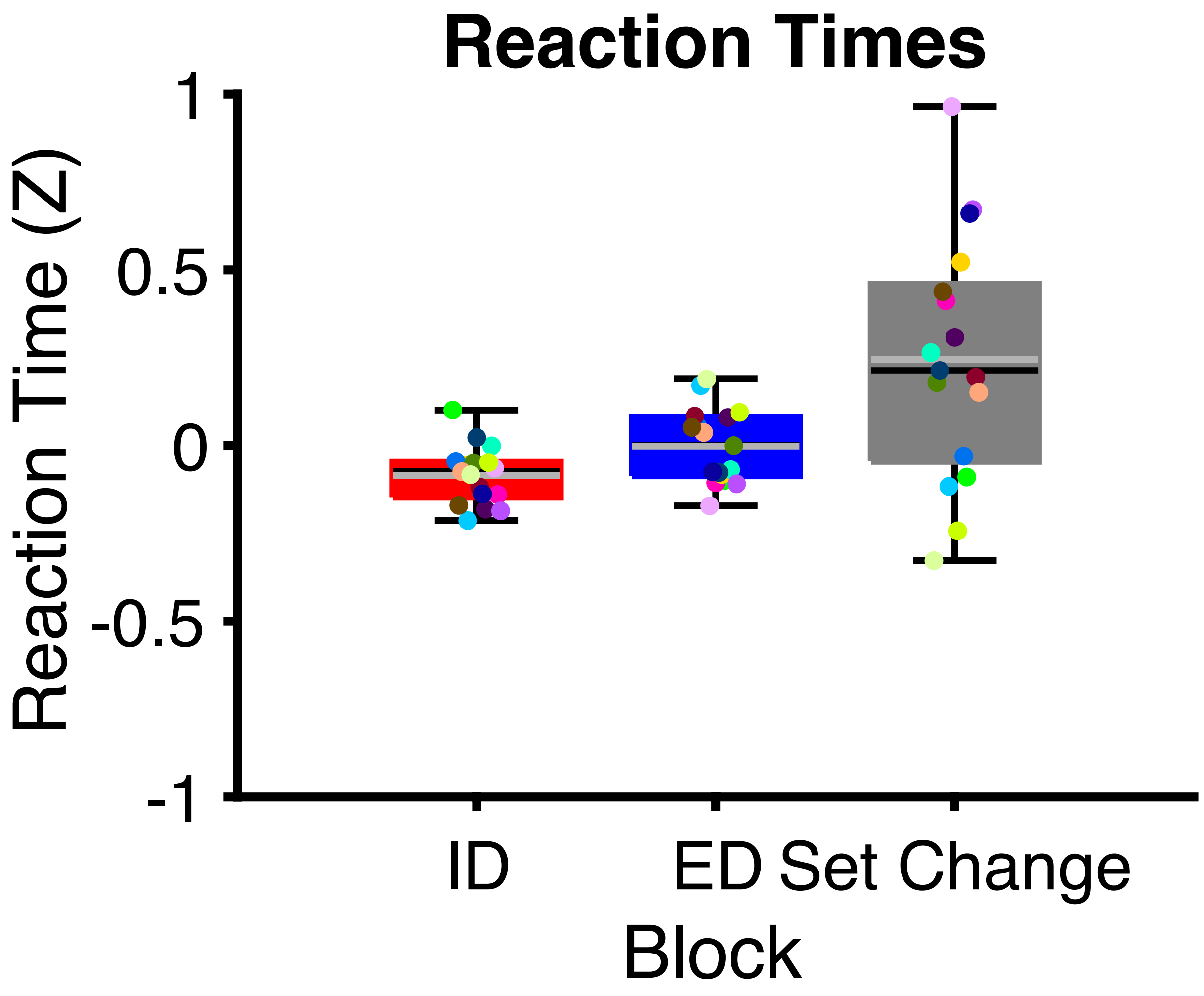

Supplementary figure 1. Reaction times when conditions are defined by block type. Boxplots showing mean and median (grey and black lines) reaction times across ED, ID and set change conditions with range (whiskers) and inter-quartile range (box). Different colored dots represent different patients (N=17). ID = Intradimensional, ED = Extradimensional. Source data are provided as a Source Data file.

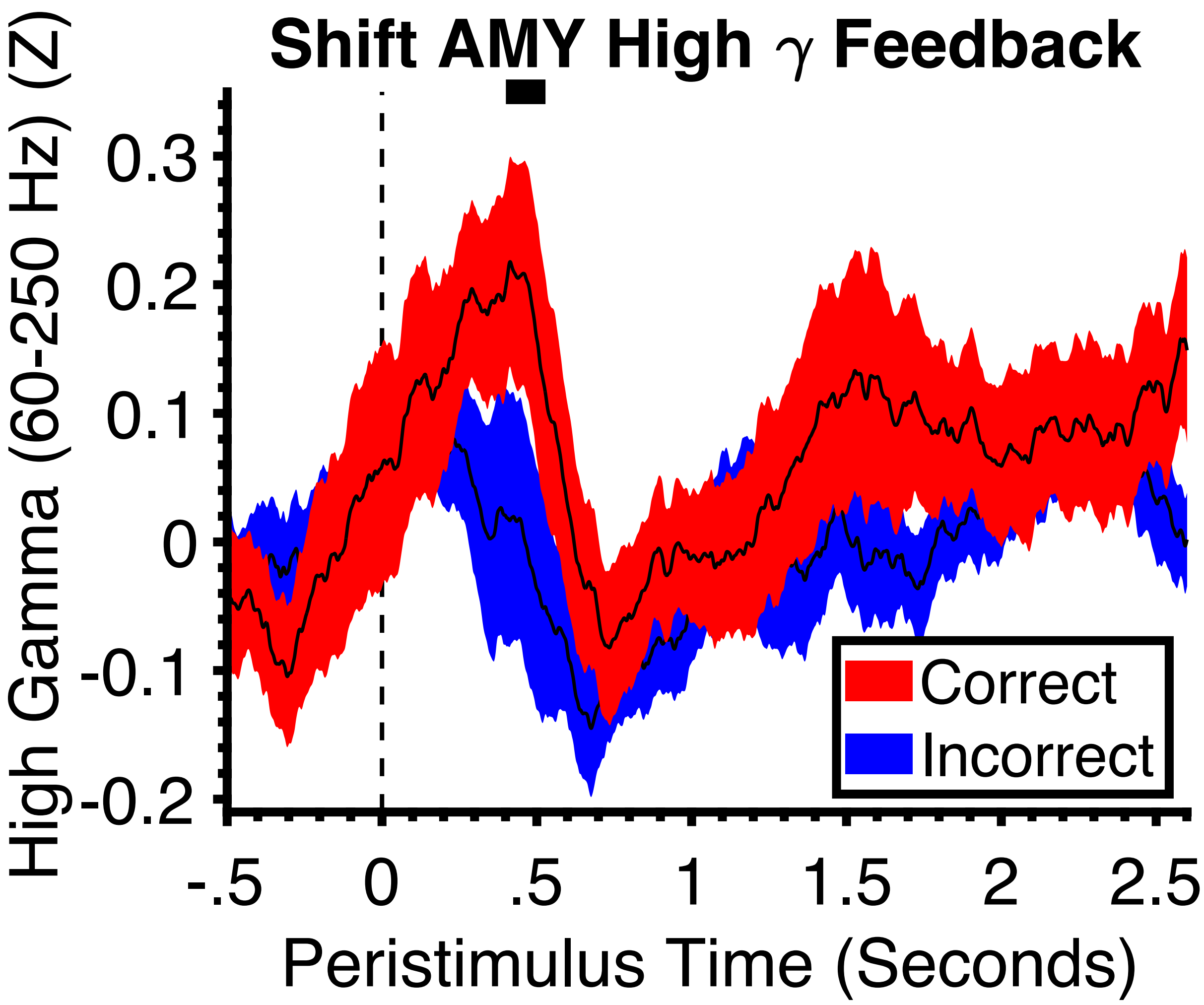

Supplementary figure 2. Amygdala high gamma responses to correct and incorrect feedback on shift trials. A. High gamma activity in the amygdala in response to correct and incorrect feedback on shift trials (shaded regions represent standard error and condition (see legend)). The horizontal black line at the top of the plot represents the time interval of cluster that approached significance (permutation test, p=.0376, FWEC, two-tailed). Vertical dashed line at t=0 corresponds to feedback stimulus onset time. The feedback stimulus was presented for 600 ms after which was the inter-trial interval/ fixation. AMY = Amygdala, ID = Intradimensional, ED = Extradimensional. Source data are provided as a Source Data file.

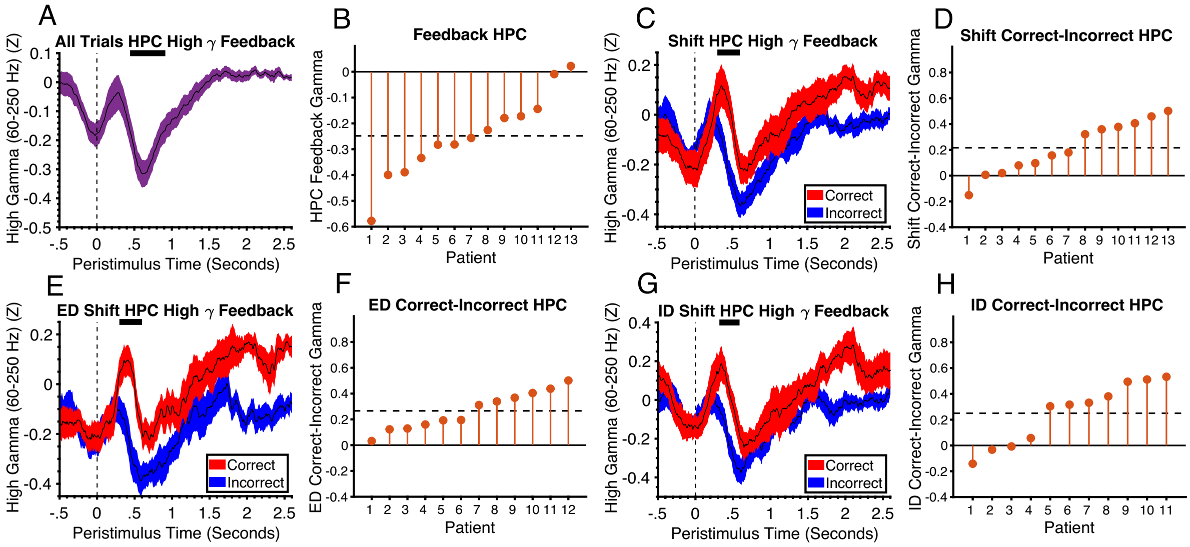

Supplementary figure 3. Hippocampal high gamma responses to correct and incorrect feedback codes win-stay. A. High gamma activity in the hippocampus in response to feedback relative to baseline across all trials (black line represents mean and shaded region represents standard error). The horizontal black line at the top of the plot represents the time intervals of significant differences from baseline (Wilcoxon's signed ranks test, $p<.025$, FDR corrected, two-tailed). Vertical dashed line at t=0 corresponds to feedback stimulus onset time. **B.** Mean activity across patients within significant time points shown in A ordered by size of effect. Horizontal dashed line is the mean difference across all patients. **C.** High gamma activity in the hippocampus in response to correct and incorrect feedback on shift trials (black lines represent mean and shaded regions represents standard error and condition (see legend)). The horizontal black line at the top of the plot represents the time intervals of significant clusters (permutation test, $p=.0061$, FWEC, two-tailed). **D.** Difference in mean activity between conditions across patients within significant time points shown in C. **E.** High gamma activity in the hippocampus in response to correct and incorrect feedback on ED shift trials (black lines represent mean and shaded regions represents standard error and condition (see legend)). The horizontal black line at the top of the

plot represents the time intervals of significant clusters (permutation test, $p<.001$, FWEC, two-tailed). **F.** Difference in mean activity between conditions across patients within significant time points shown in E. **G.** High gamma activity in the hippocampus in response to correct and incorrect feedback on ID shift trials (black lines represent mean and shaded regions represents standard error and condition (see legend)). The horizontal black line at the top of the plot represents the time intervals of significant clusters (permutation test, $p=.012$, FWEC, two-tailed). **H.** Difference in mean activity between conditions across patients within significant time points shown in G. HPC = hippocampus, ID = Intradimensional, ED = Extradimensional. Source data are provided as a Source Data file.

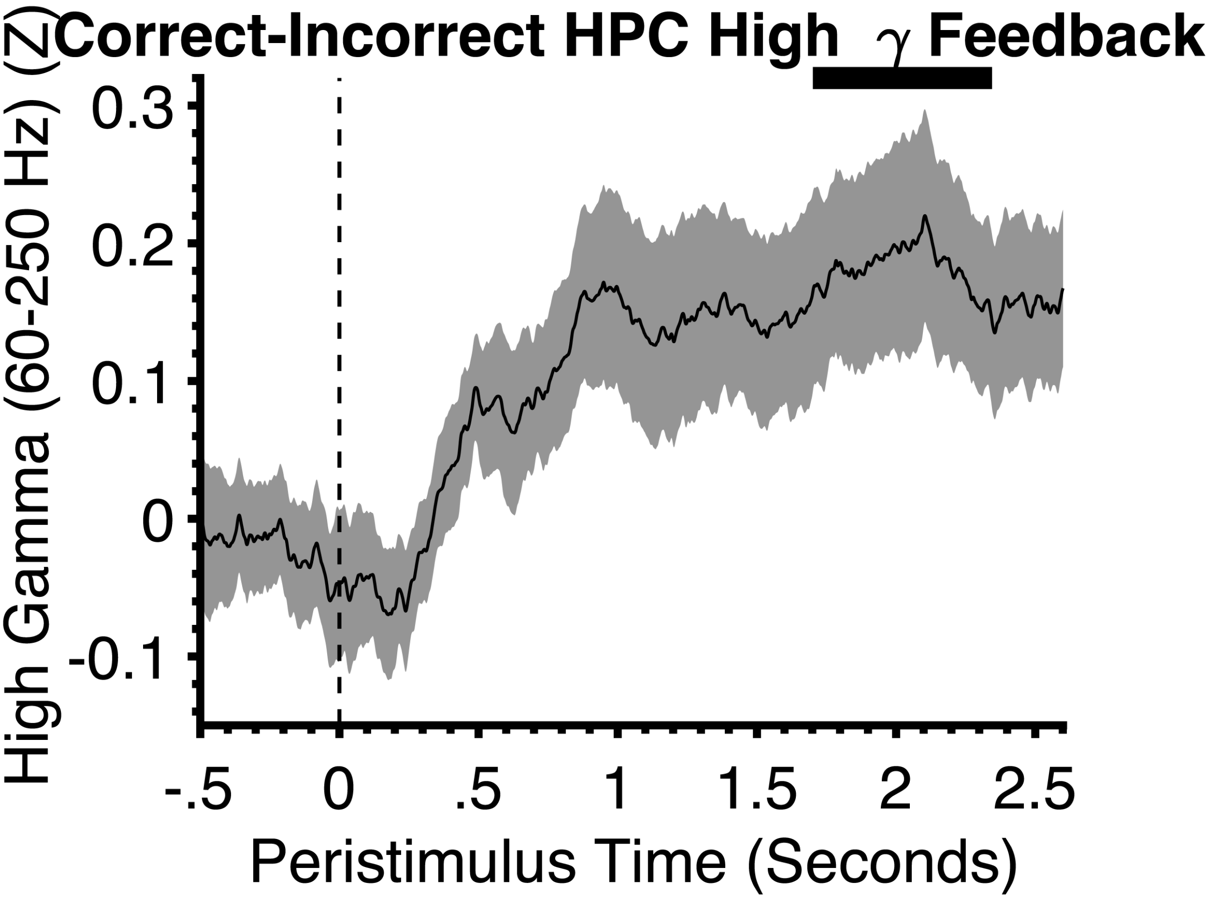

Supplementary figure 4. Difference in hippocampal high gamma activity between correct and incorrect conditions in the inter-trial-interval (ITI) emerges gradually beginning at feedback reception. Difference in high gamma activity between correct and incorrect trials (correct-incorrect) in the hippocampus in the feedback and subsequent ITI period (shaded region represents standard error of the difference). The horizontal black line at the top of the plot represents the time intervals of significant differences between regions (permutation test, p=.017, FWEC, two-tailed). Vertical dashed line at t=0 corresponds to feedback onset time. HPC = hippocampus. Source data are provided as a Source Data file.

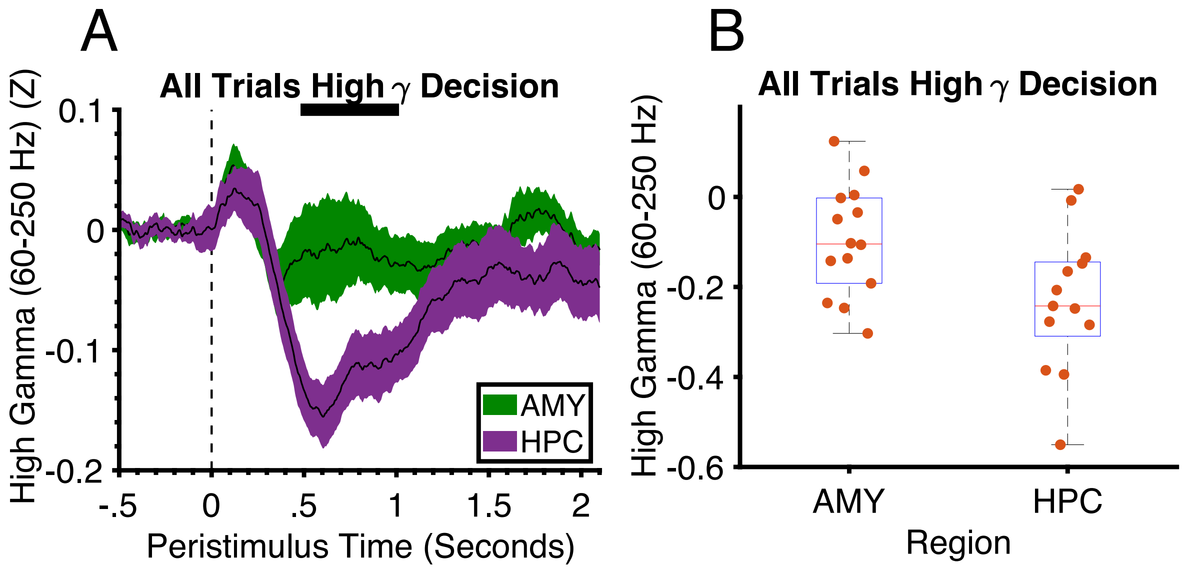

Supplementary figure 5. High gamma inhibition in the decision phase is greater in the hippocampus compared to the amygdala. A. High gamma activity in the amygdala and hippocampus in the decision phase across all trials (shaded region represents standard error and region (See legend)). The horizontal black line at the top of the plot represents the time intervals of significant differences between regions (permutation test, p=.015, FWEC, two-tailed). Vertical dashed line at t=0 corresponds to decision phase onset time **B.** Box-plots of mean activity in the amygdala and hippocampus for each patient averaged over the significant time window shown in A. Dots represent the mean of each patient. Red horizontal line shows the group mean. Box and whiskers represent inter-quartile range and range. AMY = Amygdala, HPC = hippocampus. Source data are provided as a Source Data file.

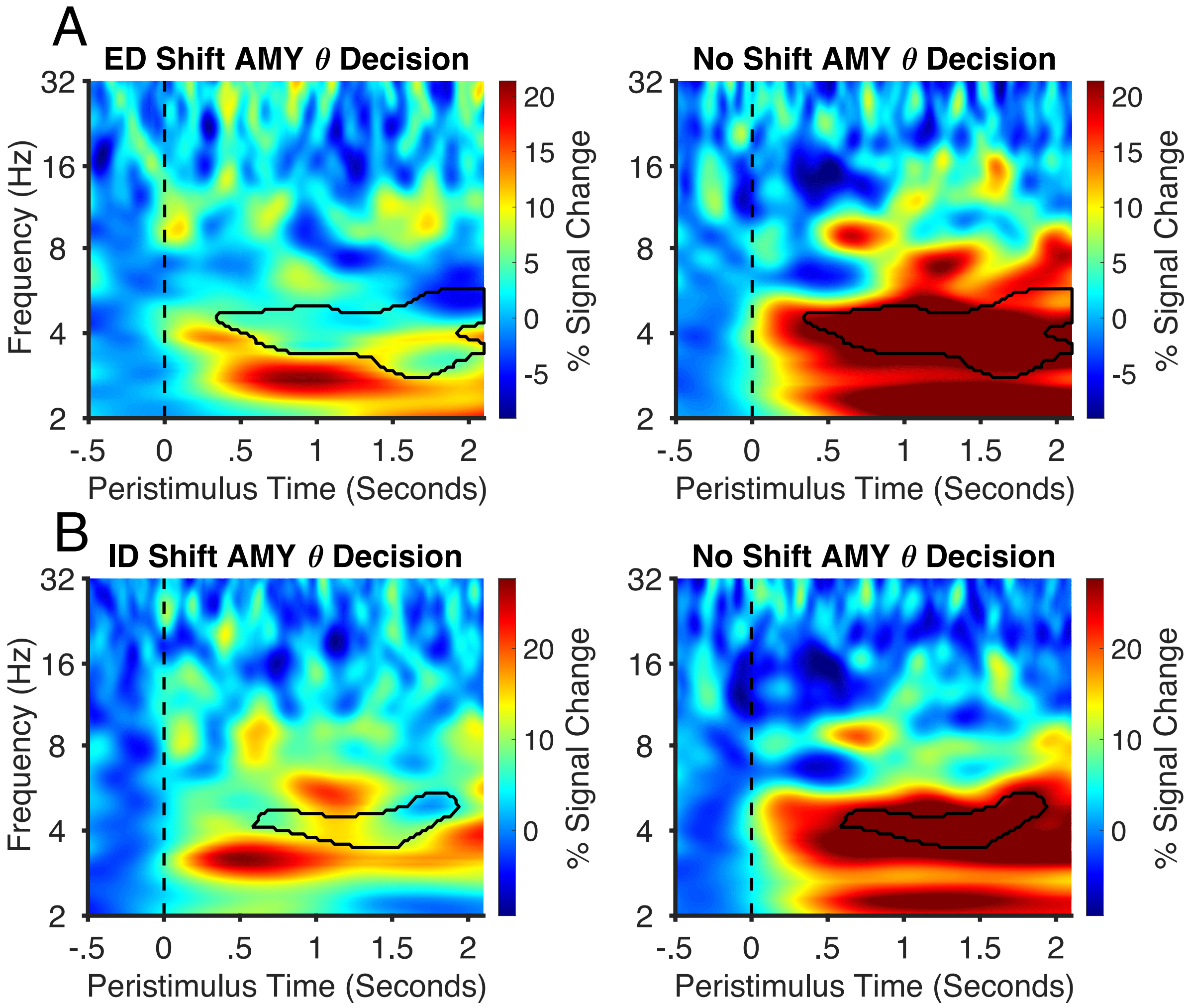

Supplementary figure 6. Theta activity in the amygdala on no shift relative to shift trials in the decision phase is driven by both ED and ID shifts. A. Time-frequency plots showing differences in theta power in the decision phase between ED shift and no shift conditions. Black outline highlights the significant cluster (permutation test, p<.001, FWEC, two-tailed). Vertical dashed line at t=0 corresponds to choice stimulus onset time. The decision phase was 2.1 seconds. **B.** Time-frequency plots showing differences in theta power in the decision phase between ID shift and no shift conditions. Black outline highlights the significant cluster (permutation test, p<.025, FWEC, two-tailed). AMY = Amygdala, HPC = hippocampus, ID = Intradimensional, ED = Extradimensional. Source data are provided as a Source Data file.

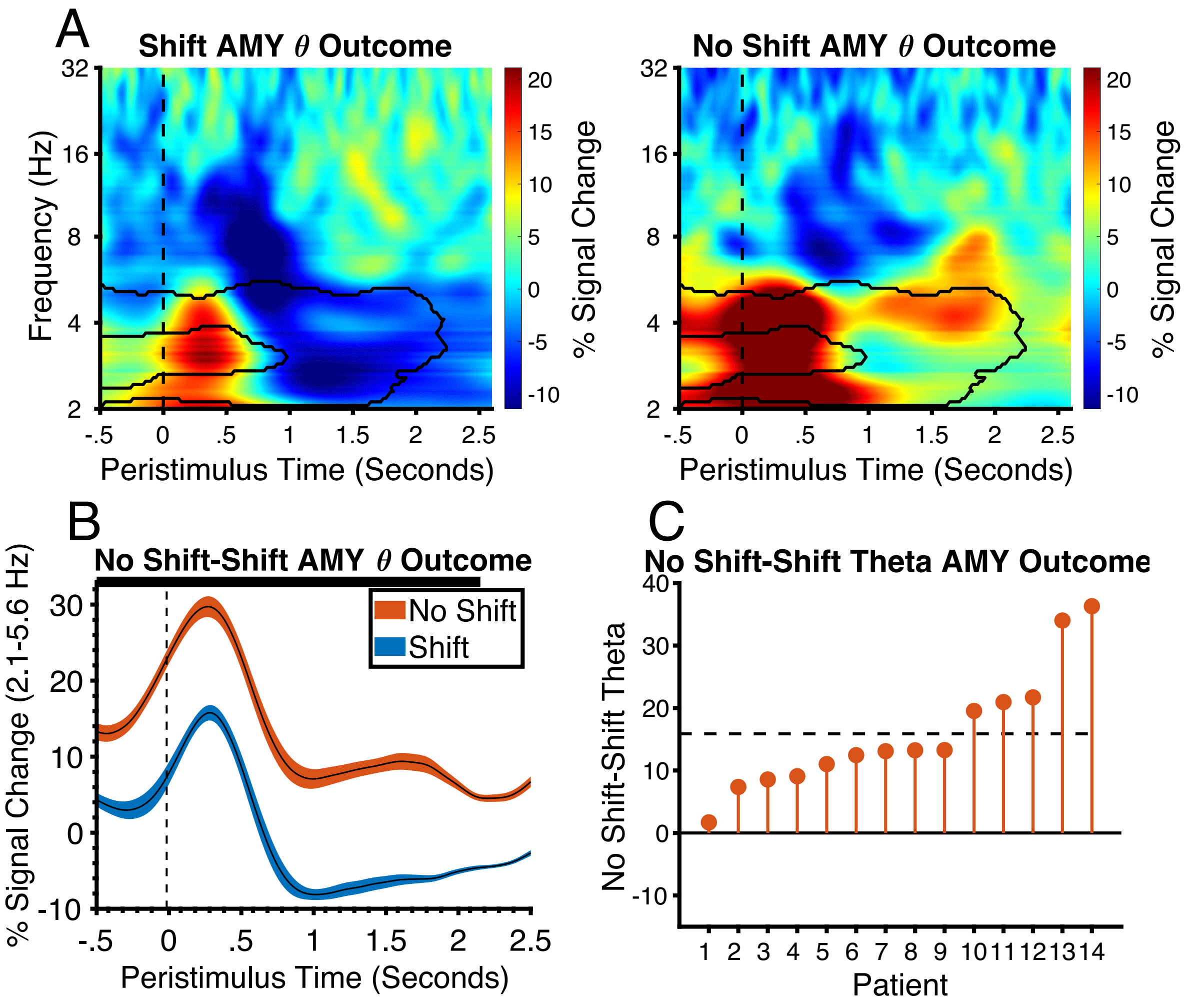

Supplementary figure 7. Amygdala theta activity is sustained into the outcome phase. A. Time-frequency plots showing differences in theta power in the outcome phase between shift and no shift conditions. Black outline highlights the significant cluster (permutation test, p<.001, FWEC, two-tailed). Vertical dashed line at t=0 corresponds to outcome stimulus onset time. The outcome phase was .6 seconds followed by the ITI for 2 seconds. **B.** Time-series of theta activity in the amygdala in the outcome phase on shift and no shift trials averaged over the frequency range spanned by the significant cluster shown in A (shaded regions represent standard error and condition membership (see legend)). The horizontal black line at the top of the plot represents the time intervals of significant clusters. **C.** Difference in mean activity between conditions across patients within significant time points shown in A and B

ordered by size of effect. Horizontal dashed line is the mean difference across all patients. AMY = Amygdala, HPC = hippocampus, ID = Intradimensional, ED = Extradimensional. Source data are provided as a Source Data file.

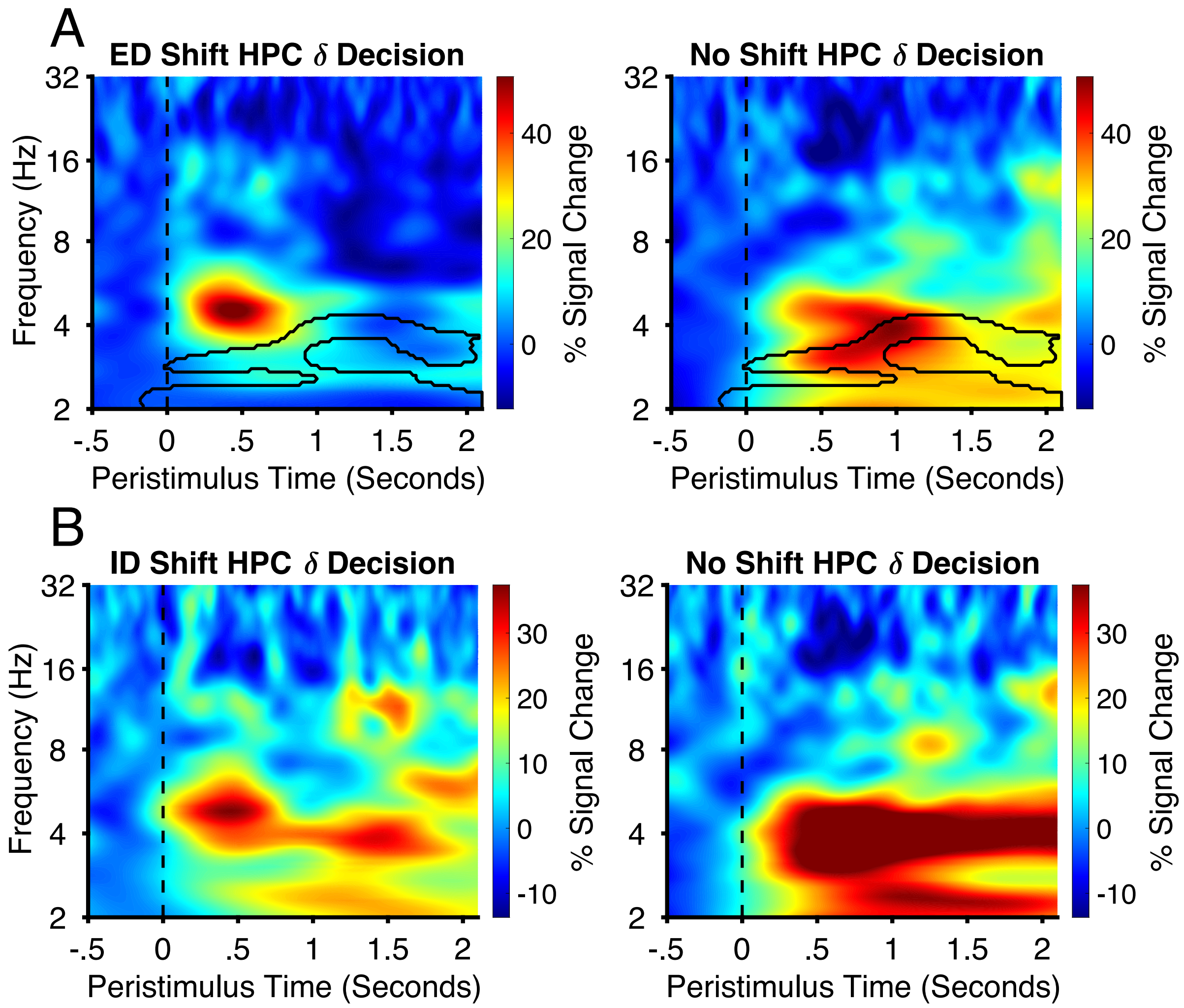

Supplementary figure 8. Low frequency hippocampal activity on no shift relative to ED and ID shift trials in the decision phase. A. Time-frequency plots showing differences in delta power in the decision phase between ED shift and no shift conditions. Black outline highlights the significant cluster (permutation test, p<.001, FWEC, two-tailed). Vertical dashed line at t=0 corresponds to choice stimulus onset time. The decision phase was 2.1 seconds. **B.** Time-frequency plots showing differences in power in the decision phase between ID shift and no shift conditions. AMY = Amygdala, HPC = hippocampus, ID = Intradimensional, ED = Extradimensional. Source data are provided as a Source Data file.

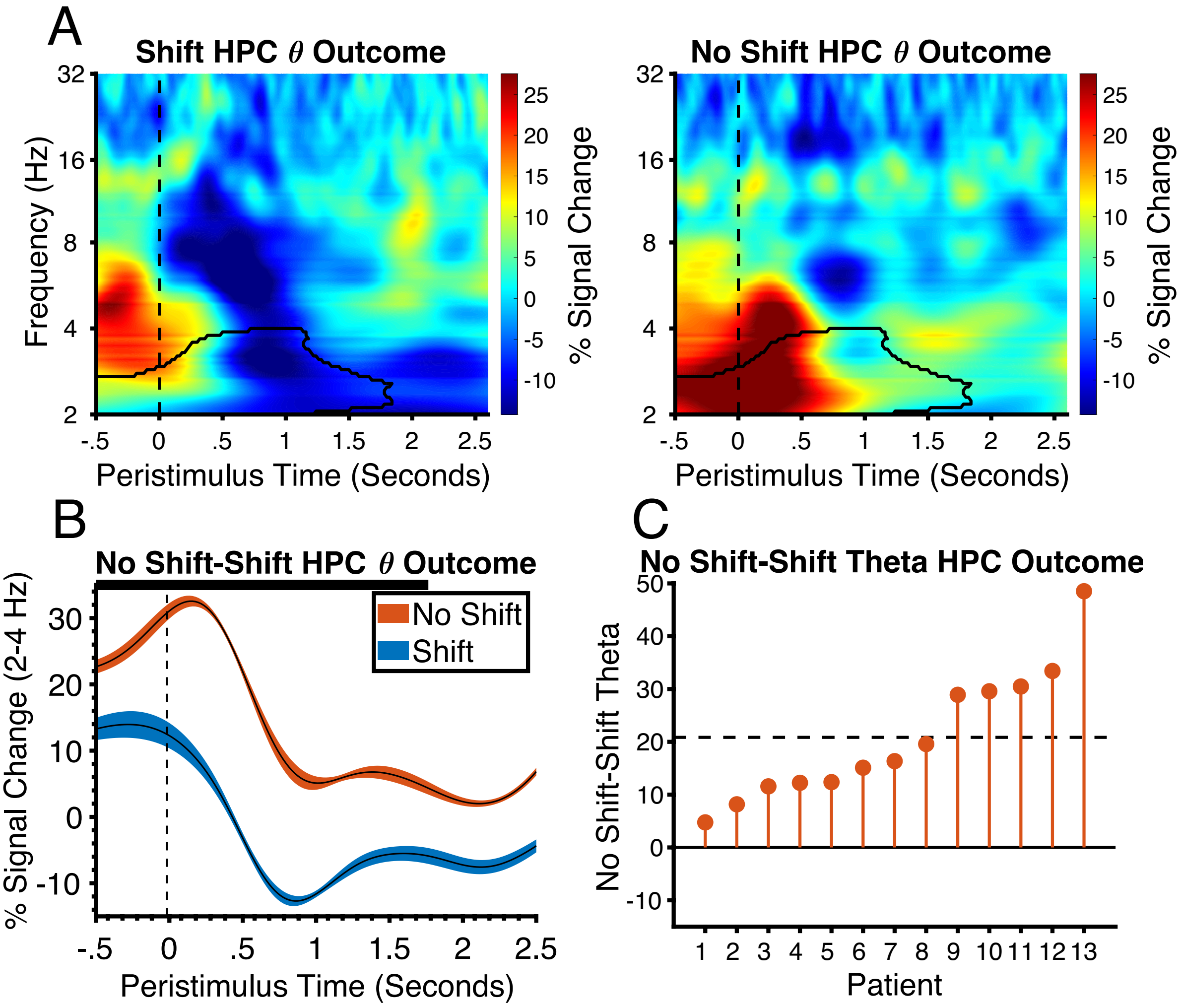

Supplementary figure 9. Hippocampus delta activity is sustained into the outcome phase. A. Time-frequency plots showing differences in hippocampus delta power in the outcome phase between shift and no shift conditions. Black outline highlights the significant cluster (permutation test, p<.025, FWEC, two-tailed). Vertical dashed line at t=0 corresponds to outcome stimulus onset time. The outcome phase was .6 seconds followed by the ITI for 2 seconds. **B.** Time-series of delta activity in the hippocampus in the outcome phase on shift and no shift trials averaged over the frequency range spanned by the significant cluster shown in A (shaded regions represent standard error and condition (see legend)). The horizontal black line at the top of the plot represents the time intervals of significant clusters. **C.** Difference in mean activity between conditions across patients within significant time points shown

in A and B ordered by size of effect. Horizontal dashed line is the mean difference across all patients. AMY = Amygdala, HPC = hippocampus, ID = Intradimensional, ED = Extradimensional. Source data are provided as a Source Data file.

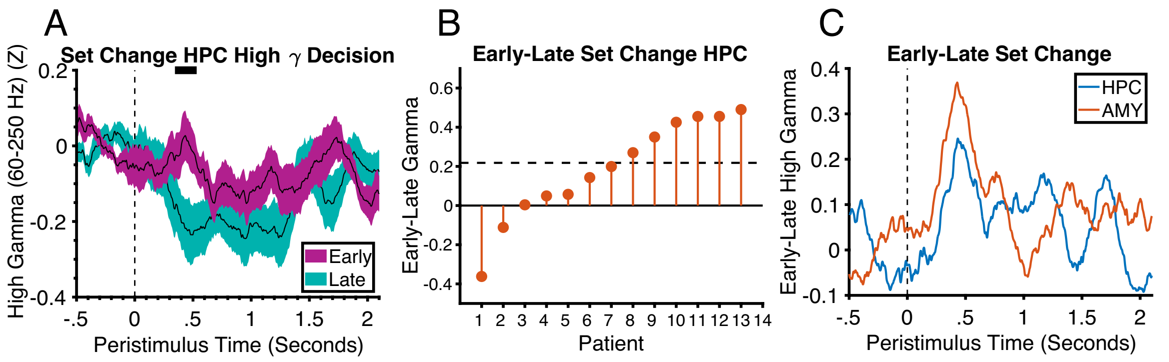

Supplementary figure 10. High gamma activity in the hippocampus during novelty processing is weaker but not significantly different compared to the amygdala. A. High gamma activity in the hippocampus in the decision phase in the first block (early/ novel) relative to the last block (late/ familiar) after a stimulus set change (shaded regions represent standard error and condition (see legend)). The horizontal black line at the top of the plot represents the time intervals of the cluster that approached significance (permutation test, p=.07, FWEC, two-tailed). Vertical dashed line at t=0 corresponds to choice stimulus onset time. The decision phase was 2.1 seconds. **B.** Mean difference in activity between conditions across patients within trending significant cluster shown in A ordered by size of effect. Horizontal dashed line is the mean difference across all patients. **C.** Difference in high gamma between early and late trials in the amygdala (orange) and hippocampus (blue) showing similar timing of the novelty effect. AMY = Amygdala, HPC = hippocampus. Source data are provided as a Source Data file.
